# Prenatal Exposure to Cannabis: Effects on Childhood Obesity and Cardiometabolic Health

**DOI:** 10.1007/s13679-023-00544-x

**Published:** 2024-01-03

**Authors:** Brianna F. Moore

**Affiliations:** 1 Department of Epidemiology, Colorado School of Public Health, Aurora, CO USA; 2 Lifecourse Epidemiology of Adiposity and Diabetes (LEAD) Center, University of Colorado Anschutz Medical Campus, 1890 N Revere Ct, Aurora, 80045 CO USA

**Keywords:** Prenatal, Cannabis, Weight, Obesity, Glucose, Metabolic

## Abstract

**Purpose of Review:**

To consolidate information on the obesogenic and cardiometabolic effects of prenatal exposure to cannabis.

**Recent Findings:**

A PubMed search strategy updated from January 1, 2014, through 14 June 2023, produced a total of 47 epidemiologic studies and 12 animal studies. Prenatal exposure to cannabis is consistently associated with small for gestational age and low birth weight. After birth, these offspring gain weight rapidly and have increased adiposity and higher glucose (fat mass percentage) in childhood. More preclinical and prospective studies are needed to deepen our understanding of whether these associations vary by sex, dose, timing, and composition of cannabis (e.g., ratio of delta-Δ9-tetrahydrocannabinol [Δ9-THC] to cannabidiol [CBD]). Addressing these gaps may help to solidify causality and identify intervention strategies.

**Summary:**

Based on the available data, clinicians and public health officials should continue to caution against cannabis use during pregnancy to limit its potential obesogenic and adverse cardiometabolic effects on the offspring.

## Introduction

Amid increasing legality and growing cultural acceptance, cannabis use in pregnancy is becoming increasingly common. Self-reported data from the 2018 National Survey on Drug Use and Health estimate that 3.7% of pregnant people use cannabis [1]. The survey data further shows a higher prevalence among pregnant people who were younger (13.1%) [1] or with a cognitive disability (13.0%) [2] or depression (12.7%) [1]. However, these estimates likely suffer from underreporting. In fact, bioanalytic data from urban hospital settings suggest that up to 30% of pregnant people use or are exposed to cannabis [3, 4].

The reasons for cannabis use vary [5]. Pregnant people report using cannabis to manage nausea, to cope with stress or anxiety, and/or for relaxation and enjoyment [6]. There are risks to the offspring that should be weighed in the decision to use cannabis during pregnancy. Prenatal exposure to cannabis was associated with adverse birth outcomes, such as lower birth weight, smaller head circumference, lower Apgar scores, and an increased risk for admission to the neonatal intensive care unit (NICU), as well as altered neurobehavioral traits among older child offspring [7–11].

More recently, evidence suggests that prenatal exposure to cannabis may predispose the offspring to obesity, altered glucose homeostasis, and impaired cardiac function [12], but there is a need to better understand this growing science. Thus, the goals of this review are to (1) establish the obesogenic and cardiometabolic risks of prenatal exposure to cannabis; (2) recontextualize the cannabis-birth weight literature with respect to childhood obesity and metabolic disorders; (3) propose biological mechanisms underlying these associations; (4) compare the effects of common cannabinoids, namely, delta-Δ9-tetrahydrocannabinol (Δ9-THC) and cannabidiol (CBD); and (5) highlight critical gaps in knowledge that are needed to infer causality and identify opportunities for intervention.

## Methods

A PubMed search strategy was updated through 14 June 2023. The search algorithm included all possible combinations of keywords from the following three groups: (1) “cannabis,” “marijuana,” “THC,” or “CBD”; and (2) “pregnancy,” “prenatal,” “in utero,” “fetal,” “offspring,” “infant” or “early life”; and (3) “birth outcomes,” “birth weight,” “weight,” “obes*,” “body mass index,” “fat mass,” “adipo*,” “glucose,” “insulin,” “metabolic syndrome,” “cardio*,” “cardiac,” “blood pressure,” “cholesterol,” “hypertension,” or “lipid.” This review focused on papers published since 2014, given the increasing legality of cannabis for recreational use over the past decade and changes in cannabis potency [13].

## Results

The PubMed search identified 1898 publications. After removing duplicates, 491 unique publications were screened. Of these, 432 were excluded for the following reasons: commentaries or reviews (*n* = 71); neurodevelopment, cognitive, or behavioral outcomes (*n* = 72); other unrelated health outcomes (*n* = 86); studies of hemp oil, synthetic or endogenous cannabinoids, or the endocannabinoid system (*n* = 36); polysubstance use or drugs other than cannabis (*n* = 24); characteristics or reasons for cannabis use (*n* = 43); prevalence studies (*n* = 18); biomarker studies (*n* = 16); cessation or prevention studies (*n* = 9); health policy analyses (*n* = 3); effects of pre-conception cannabis use (*n* = 3); abstracts or reports presenting insufficient data (*n* = 2); and papers focused entirely on unrelated exposures and outcomes (*n* = 50). Thus, 47 epidemiologic studies [3, 4, 14–18, 19••, 20–28, 29•, 30–33, 34••, 35–58] and 12 animal models [59, 60••, 61, 62, 63••, 64, 65••, 66•, 67–70] presenting original data met the inclusion criteria for this review.

### Evidence from Human Studies

Table 1 summarizes the 47 human epidemiologic studies [3, 4, 14–18, 19••, 20–28, 29•, 30–33, 34••, 35–58]. The most common study design was a retrospective medical record review, followed by a prospective cohort study. Cannabis exposure was most often ascertained through self-report (*n* = 22; 47%), though many studies measured Δ9-THC in maternal urine collected in pregnancy (*n* = 17; 36%). Four studies captured exposure through Δ9-THC detected in meconium or umbilical cord tissue homogenate collected at delivery (9%). Cannabis use disorder was the primary exposure of interest in three studies (6%). The prevalence of cannabis use or exposure during pregnancy in the absence of other substances ranged from 2.0 to 38.9%. Co-use of cannabis and other drugs of abuse is common. In a case-control study, Kong et al. [29•] reported that co-use of tobacco and cannabis in pregnancy was nearly 41.3%. Three studies estimated that cannabis use among opioid-dependent pregnant people ranged from 9.4% [40] to 40.0% [36].

**Table 1:**
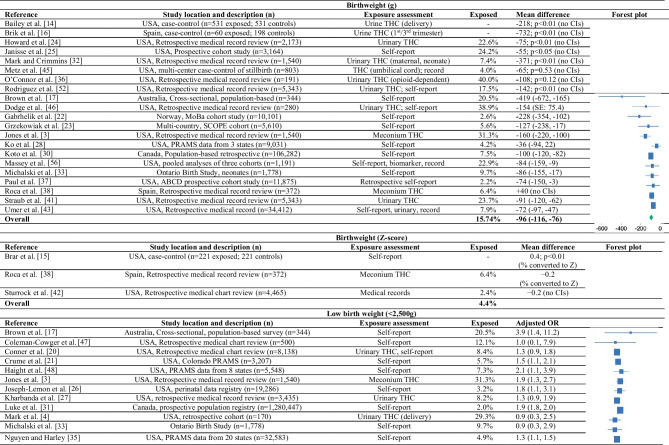

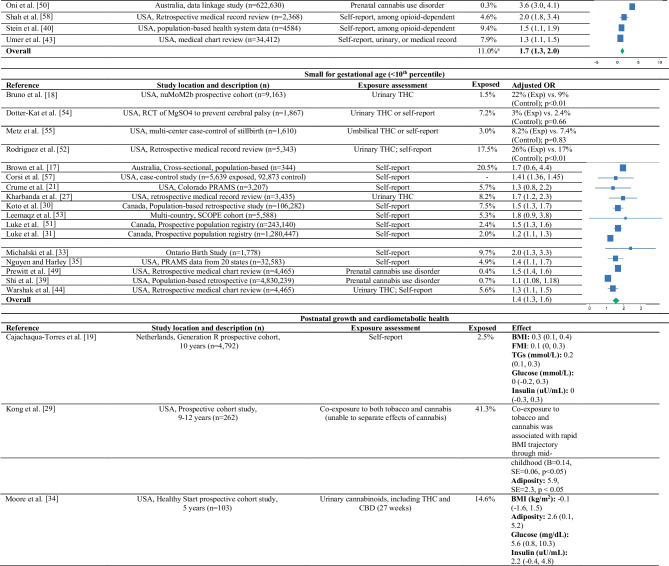
Human studies on prenatal exposure to cannabis and offspring birth weight, postnatal growth, adiposity, and cardiometabolic health

*BMI* body mass index, *CBD* cannabidiol, *FMI* fat mass index, *MoBa* Mother and Child Cohort Study, *PRAMS* Pregnancy Risk Assessment Monitoring System, *RCT* randomized controlled trial, *SCOPE* Screening for Pregnancy Endpoints, *TGs* triglycerides, *THC* Δ9-tetrahydrocannabinol

Forty-four studies examined the associations between prenatal exposure to cannabis and birth weight (as a continuous measure), low birth weight (< 2500 g), or small for gestational age (SGA; < 10th percentile). Prenatal exposure to cannabis was associated with a 55 to 732 g reduction in birth weight, with most studies (17 out of the 21) showing a statistically significant reduction in birth weight, independent of gestational age and sex. Roca and colleagues [38] reported that prenatal exposure to cannabis was associated with a 40 g increase in birth weight among NICU-admitted neonates. However, consistent with findings by Sturrock and colleagues [42], they reported that prenatal exposure to cannabis was associated with a lower birth weight percentile. No effect was noted in a retrospective medical record review of opioid-dependent pregnant people [36], in a population-based case-control study of stillbirths [45], or in a cross-sectional analysis of Pregnancy Risk Assessment Monitoring System (PRAMS) data [28].

Prenatal exposure to cannabis was associated with a statistically significant increase in odds of LBW (adjusted odds ratios [aORs] ranging from 0.9 to 3.9; 11 out of 16 studies showing statistical significance) and SGA (aORs ranging from 1.1 to 2.0; 12 of 17 studies showing statistical significance). The effects were generally independent of gestational age, sex, tobacco/substance in pregnancy, and socioeconomic covariates, such as household income or maternal education. However, the potential for confounding remains moderate. Two studies presented unadjusted models [32, 36], few studies adjusted for maternal pre-pregnancy body mass index (BMI) or gestational weight gain, and none adjusted for maternal diet in pregnancy.

Only one published study has examined the association between prenatal exposure to cannabis and postnatal growth. Using data from a New York–based prospective cohort, Kong et al. [29•] reported that co-exposure to tobacco and cannabis was associated with rapid BMI growth from birth through mid-childhood. However, the effects of cannabis could not be isolated from the effects of tobacco. In an unpublished manuscript, I (along with my co-authors) showed that prenatal exposure to cannabis was associated with rapid BMI growth from birth through age 3 years in the Healthy Start study. Together, these studies suggest that cannabis-exposed infants are smaller at birth, grow rapidly in infancy, and exceed the BMI of unexposed offspring by ~9 months of age. This pattern of growth is often associated with an increased risk for obesity [71], metabolic syndrome [72], and type 2 diabetes [73] later in life.

In the Generation R Study, Cajachagua-Torres and colleagues [19••] reported that maternal or paternal self-report of cannabis use during pregnancy was associated with higher triglycerides and BMI among 10-year-old offspring. This finding is supported by the work of Moore et al. [34••] and Kong et al. [29•], which reported higher adiposity (fat mass percentage) among Δ9-THC-exposed children. However, there is some uncertainty around the effects on metabolic outcomes. Cajachagua-Torres and colleagues [19••] reported no association with non-fasting glucose. Conversely, Moore and colleagues [34••] showed that prenatal exposure to cannabis was associated with increased fasting glucose at age 5 years.

### Evidence from Animal Studies

Table 2 summarizes the 12 animal studies included in this review [59, 60••, 61–70]. Wistar rats were the most common model, followed by C57BL/6 J mice and Sprague-Dawley rats. All but one study included both sexes in their experiments. The route of exposure varied, with most experiments utilizing intraperitoneal injection or oral administration. Exposure typically occurred between 6 and 22 days gestation, though some studies included exposure during mating and through weaning.

**Table 2:** Animal studies of prenatal exposure to cannabinoids and offspring growth, adiposity, and cardiometabolic health

**Birth weight**							
**Reference**	**Cannabinoid**	**Model**	**Administration**	**Timing**	**Dose (per day)**	***N* (exp, con)**	**Effect**
Benevenuto et al. [69]	THC	Balb/C mice	Ambient	GD 5.5–17.5	5 min of smoke	37, 30	Lower BW, stronger effects in males
Breit et al. [59]	THC	Sprague-Dawley rats	Ambient	GD 20	30 min of smoke	13, 13	No difference in BW
Gillies et al. [60••]	THC	Female Wistar rats	Intraperitoneal injection	GD 6–birth	3 mg/Kg	5, 6	Females: Lower BW
Lallai et al. [62]	THC	Wistar rats	Oral administration	GD 1–20	5 mg/Kg	11, 10	Lower BW
Lee et al. [63••]	THC	Wistar rats	Intraperitoneal injection	GD 6–22	3 mg/Kg	8, 8	Lower BW, lower heart-to-BW ratio
Natale et al. [64]	THC	Wistar rats	Intraperitoneal injection	GD 6–22	3 mg/Kg	8, 8	Lower BW, lower liver-to-BW ratio
Oke et al. [65••]	THC	Wistar rats	Intraperitoneal injection	ED 6.5–22	3 mg/Kg	8, 8	Lower liver-to-BW ratio
Robinson et al. [66•]	THC	C57BL/6 J mice	Oral administration	ED 3.5–12.5/17.5	5 or 10 mg/kg	36, 31, 35	Lower fetal weight at ED 17.5

**Postnatal growth and cardiometabolic outcomes**							
Asadi et al. [68]	THC	Wistar rats	Intraperitoneal injection	GD 6–birth	3 mg/Kg	8, 8	Females: Increased glucagon-to-insulin ratio (21 days) No difference in glucose or insulin
Iezzi et al. [61]	CBD	C57BL/6 J mice	Subcutaneous injection	GD 5–18	3 mg/Kg	14, 18	Males: Higher postnatal weight (10–22 days) Females: No difference in postnatal weight
Wanner et al. [67]	CBD	Female A^vy^ mice	Oral administration	Mating-weaning	20 mg/Kg	23, 26	No difference in postnatal weight (12 weeks)
Oke et al. [65••]	THC	Wistar rats	Intraperitoneal injection	ED 6.5–22	3 mg/Kg	8, 8	Increased adiposity (6 months) Males: Higher hepatic triglycerides (6 months)
Gillies et al. [60••]	THC	Female Wistar rats	Intraperitoneal injection	GD 6–birth	3 mg/Kg	5, 6	No difference in postnatal weight (12 days, 5 months) Females: Glucose intolerance, decreased pancreatic B-cell mass (5 months)
Lallai et al. [62]	THC	Wistar rats	Oral administration	GD 1–20	5 mg/Kg	11, 10	No difference in postnatal weight (15 days)
Lee et al. [63••]	THC	Wistar rats	Intraperitoneal injection	GD 6–22	3 mg/Kg	8, 8	Lower stroke volume, lower cardiac output, adverse left ventricular function (3 weeks)
Maciel et al. [70]	THC, CBD	CD1 mice			3 mg/Kg	11, 14, 13	No difference in weight at 21 or 60 days
Robinson et al. [66•]	THC	C57BL/6 J mice	Oral administration	ED 3.5–12.5/17.5	5 or 10 mg/Kg	36, 31, 35	Myocardial hyperplasia, semilunar valve thickening, lower body/heart weight (21 days)

*BW* birth weight, *ED* embryonic day, *GD* gestational day, *PD* postnatal day

The doses administered to the rodents ranged from 2 to 10 mg/Kg of Δ9-THC per day and 3 to 20 mg/Kg of CBD per day. This dose mimics moderate recreational cannabis use in human adults (13 to 63 ng/mL), which results in a fetal dose between 4 and 287 ng/mL [60••].

Seven of the eight animal studies reported lower birth weights among offspring with prenatal exposure to Δ9-THC. Breit and colleagues [59] found no association, though the Sprague-Dawley rats were exposed only once (30 min of cannabis smoke at gestational day 20). This may provide insight about how frequency and dose may influence offspring birth weight and later-life adiposity.

Both Δ9-THC and CBD appear to impact postnatal growth. Compared to non-exposed offspring, Δ9-THC-exposed offspring are born smaller, “catch-up” in as little as 12 days [60••, 62], and begin to surpass the size of non-exposed offspring by age 6 months [65••]. Prenatal CBD may impact postnatal growth in a sex-specific manner, with males being more susceptible to this environmental insult [61, 67], but the evidence is not conclusive [70].

Prenatal exposure to Δ9-THC impacts many aspects of offspring’s cardiovascular and metabolic health. Robinson and colleagues [66•] demonstrated that C57BL/6 J mice with prenatal exposure to Δ9-THC had myocardial valve thickening and ventricular septal defect. These structural changes to the fetal heart may have long-lasting impacts on cardiovascular function. Indeed, Lee and colleagues [63••] demonstrated that prenatal Δ9-THC was associated with lower stroke volume and cardiac output. Additional animal studies conducted at the University of Western Ontario revealed that prenatal Δ9-THC was associated with increased glucose intolerance at age 5 months among female offspring only [60••] and higher hepatic triglycerides at age 6 months among male offspring only [65••]. Taken together, studies provide some evidence that prenatal exposure to Δ9-THC may predispose offspring to dyslipidemia and hyperglycemia across the life course.

## Discussion

### Biological Mechanisms

**Fig. 1:**
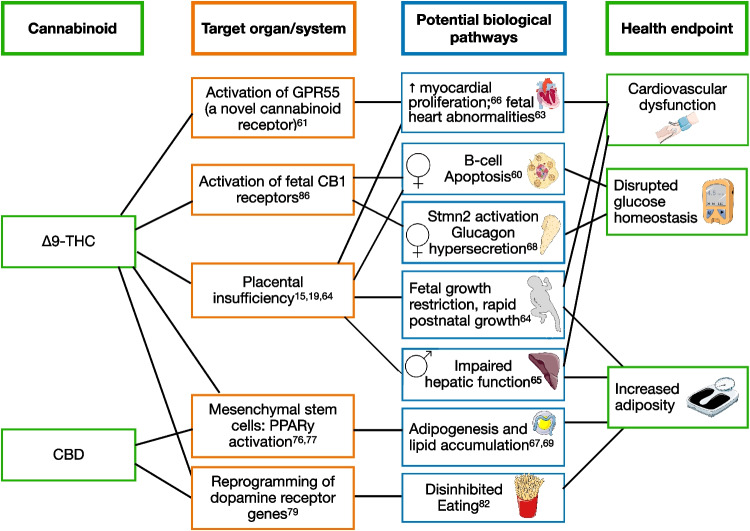
Potential mechanisms underlying the associations between prenatal exposure to cannabinoids with child obesity and cardiometabolic health. Images were obtained from the free medical site http://smart.servier.com/ by Servier licensed under a Creative Commons Attribution 3.0 Unported License

An important tenet of the causal inference framework is to establish the biological plausibility of the observed associations. Figure 1 summarizes the possible biological mechanisms linking prenatal exposure to cannabis with offspring obesity, cardiovascular health, and metabolic disorders.

Preclinical studies show that Δ9-THC leads to placental insufficiency [15, 64, 74], which may hinder fetal growth. When fetal growth restriction is followed by rapid infant growth, the offspring may be predisposed to abdominal obesity, type 2 diabetes, and cardiovascular disease [75]. However, Metz and colleagues [45] theorized that the adverse birth effects induced by prenatal exposure to cannabis may be independent of abnormal placental pathology. Thus, other mechanisms may be involved.

Fetal programming in mesenchymal stem cells (MSCs) may play an important role in the cannabis-obesity association. Both Δ9-THC [76] and CBD [77] promote adipogenesis in human and mouse MSCs via peroxisome proliferator–activated receptor gamma (PPAR***γ***) activation. Excessive adipogenesis of fetal MSCs may contribute to obesity later in life, regardless of offspring birth weight [78].

Cannabinoids may alter RNA regulation of dopamine receptor genes, as demonstrated by DiNieri et al. [79] and proposed by de Almeida and Devi [80]. Dysregulation of this key reward pathway may alter appetite and satiety and contribute to disinhibited eating. Murine models further showed that Δ9-THC-exposed male, but not female, offspring impacts mesolimbic dopamine function [81, 82]. This may explain the sex-specific effects of CBD on postnatal growth, as reported by Iezzi and colleagues [61].

The endocannabinoid system may be integral to the development of the pancreas. The endocannabinoid system is a complex signaling pathway involved in brain development [83], metabolism [84], and glucose homeostasis [85]. Cannabinoid type 1 (CB_1_) receptors are abundant in both glucagon-producing α-cells and insulin-producing β-cells [86]. In a mouse model, Malenczyk and colleagues [87] showed that 2-arachidonoylglycerol (2-AG, an endocannabinoid that is functionally similar to Δ9-THC) influenced islet morphology by increasing the number of pancreatic α-cells, which would promote glucagon release, oppose insulin action, and increase blood glucose in exposed pups. In a separate mouse model, Gillies and colleagues [60••] reported that prenatal exposure to Δ9-THC was associated with a significant reduction (41%) of pancreatic β-cells and increased glucose intolerance in female Wistar rats at a postnatal age of 5 months. Asadi and colleagues [68] provide compelling evidence that prenatal exposure to Δ9-THC reprograms fetal islet endocrine hormone profile among female rat offspring. Specifically, stathmin-2 (Stmn2) may play a role in regulating offspring glucose through its interaction with glucagon. Taken together, these studies illustrate how prenatal exposure to cannabis may disrupt the balanced molecular control of insulin and glucagon release via the endocannabinoid system.

### Individual Cannabinoids

Cannabis is a complex mixture of over 100 cannabinoids [88]. Δ9-THC and CBD are the most abundant and most studied cannabinoids. Disentangling the effects of Δ9-THC and CBD may have important health implications. Over the past two decades, there has been a dramatic shift in the composition of commercial cannabis products. Δ9-THC potency has increased three-fold, while the concentration of CBD has been halved [13]. CBD use is often perceived as safe among pregnant people and even some obstetrics-related medical professionals [89], which may explain why one in five pregnant people report CBD use while pregnant [90].

While structurally similar, Δ9-THC and CBD have different molecular targets. As such, they produce distinct and sometimes opposing effects. For instance, Δ9-THC is widely accepted as orexogenic [91], whereas CBD is often associated with reduced appetite and weight loss [92]. To date, there is very little published data regarding the metabolic effects of individual cannabinoids. This is almost certainly due to the scheduling status of the drug, which restricts access to cannabis for research purposes. A 1974 randomized controlled trial reported that a single intravenous administration of Δ9-THC (6 mg) induced glucose intolerance among healthy adult volunteers [93]. Case studies from 1969–1970 further showed that higher doses of Δ9-THC can lead to glycosuria [94] and diabetic ketoacidosis [95]. By contrast, a recent clinical trial shows that a 10:1 ratio of CBD to Δ9-THC (100 µg CBD and 10 µg Δ9-THC) improves the lipid profile and glycemic control after 8 weeks among patients with type 2 diabetes [96]. Several clinical trials are underway examining the metabolic impacts of Δ9-THC or CBD administration (e.g., NCT05322213, NCT05618756, NCT04114903), which may further elucidate the metabolic effects of Δ9-THC and CBD.

Despite this growing body of evidence, it is not clear whether prenatal exposure to Δ9-THC and CBD would produce similar cardiometabolic effects on the offspring or whether these cannabinoids impose opposite effects, as it does in adult active users. Furthermore, there are other common cannabinoids that may influence glycemic control. For instance, a recent double-blind randomized controlled trial showed that tetrahydrocannabivarin (THCV) decreased fasting plasma glucose and improved pancreatic β-cell function in adult patients with type 2 diabetes [97]. Large prospective cohorts with sufficiently large subgroups of offspring with prenatal exposure to Δ9-THC, CBD, and other common cannabinoids are needed to explore this question more conclusively in humans.

### Windows of Susceptibility

Timing may be an important factor in the associations between prenatal exposure to cannabis and offspring adiposity and cardiometabolic health. For instance, early gestation exposure may alter pancreatic development [87], whereas late gestation exposure, when the majority of fat accretion occurs [98], may have a more profound effect on birth weight and child adiposity. Few studies have attempted to examine whether early vs. late gestation exposure impacts offspring birth outcomes. Three epidemiologic studies reported no effect on birth weight or child metabolic health if the mother quit cannabis early in the pregnancy [19••, 23, 37]. A fourth study reported that cannabis use in the first trimester alone was associated with offspring birth weight, though the effects were more severe if cannabis use was sustained throughout the entire pregnancy [46]. Given this paucity of data, there remains a need to quantify exposure at multiple time points throughout pregnancy to formally assess trimester-specific effects.

Beyond the prenatal period, pre-conception exposure may predispose offspring to later-life obesity and cardiometabolic disease, but the evidence is inconsistent. Two human epidemiologic studies reported that pre-conception exposure to cannabis was associated with lower birth weight [99, 100]. This may occur through the disruption of oocyte maturation [101] or through epigenetic changes to sperm among paternal cannabis users [102]. However, this hypothesis is inconsistent with a preclinical study, which found no effect on birth weight among offspring of male Wistar rats exposed to Δ9-THC while mating [103]. More research is needed to clarify whether the epidemiologic findings are due to pre-conception exposure alone or rather a continuation of cannabis use in early pregnancy.

Childhood exposure to cannabis is also a growing concern. Self-reported data from the 2015 National Survey on Drug Use and Health estimates that 12% of adults with children in the home use cannabis [104]. Bioanalytical studies confirm that at least this many children are exposed, though the prevalence may be higher in younger children. Prospective data from a Colorado-based study indicates that 13% of children, aged 5 years, had detectable levels of CBD in urine [105]. Cross-sectional data from Denver, CO [106] and New York City, NY [107] suggest that nearly 20% of children under 3 years of age had detectable Δ9-THC concentrations in urine. Given the age of these children, it is not clear whether these exposures occur through breast milk (in which Δ9-THC readily accumulates [108]), through dermal exposure (as cannabinoids are known to accumulate on surfaces [109] and young children exhibit increased hand-to-mouth activity [110]), or through ambient exposure (an important route of exposure given children’s faster ventilation rates [111]).

### Mode, Dose, and Frequency of Use

Bioavailability varies widely based on the mode of cannabis use. Cannabinoids exhibit similar pharmacokinetics when administered via inhalation or intravenous injection: peak plasma concentrations occur rapidly (under 10 min) [112] and bioavailability is moderate (10–35%) [113]. Due to hepatic first-pass metabolism, the bioavailability of orally consumed Δ9-THC is very poor, < 20% for edible gelatin capsules and as low as 6% for baked goods [114]. As such, oral administration results in lower and more erratic plasma concentrations [112]. Given that edibles are the second most common mode of administration among pregnant people [115], it may be important to consider the mode of use in future studies examining the cardiometabolic effects of prenatal exposure to cannabis.

Few studies have evaluated potential dose-response effects, which represents an important gap in knowledge. Robinson and colleagues [66•] showed clear dose-dependent effects for 5 and 10 mg/kg Δ9-THC per day. Frequency of use may also impact these associations. Two epidemiologic studies have reported that the cannabis-birth weight association was only evident among frequent users but not among infrequent users (less than once a month) [48, 116]. Additional dose-response studies are needed to identify threshold effects and solidify the casual nature of these associations.

### Sex-Specific Effects

The published literature provides some evidence of sexually dimorphic associations between prenatal exposure to cannabinoids and offspring cardiometabolic health, though the patterns and mechanisms are not yet clear.

Males appear to be more susceptible to cannabis-induced impacts on growth. In a longitudinal epidemiologic analysis, Massey and colleagues [56] reported that prenatal exposure to cannabis was associated with lower birth weight among male offspring, but not female offspring. This observation is supported by the fact that males are generally more susceptible to early-life environmental insults [117]. Furthermore, in a mouse model, Benevenuto and colleagues [69] showed that prenatal exposure to cannabis smoke was associated with a lower fetal-placental weight ratio in male rat offspring only, which may imply sex-specific placental insufficiency.

Prenatal CBD appears to have little effect on postnatal growth, though there is some indication of a sex-specific effect. Iezzi and colleagues [61] reported that male offspring with developmental exposure to CBD had increased postnatal weights, whereas both Iezzi et al. [61] and Wanner et al. [67] reported no difference in postnatal weight among female offspring, and Maciel et al. [70] reported no difference in either sex. Sex-specific weight differences may be due to increased bioaccumulation of CBD among males [70] or CBD potentially exaggerating differences in postnatal growth trajectories among boys and girls [118].

Prenatal Δ9-THC has been linked to increased glucose intolerance among female offspring only [60••], which may be attributable to sex-specific differences in the development of the endocannabinoid system [119] (which plays a key role in metabolism [84] and glucose homeostasis [85]) or insulin resistance (which tends to be higher in prepubertal girls as compared to prepubertal boys [120]). On the other hand, prenatal Δ9-THC has also been linked to higher hepatic triglycerides among male offspring only [65••]. Oke and colleagues [65••] further showed that male, but not female, rat offspring exhibit decreased expression of miR-203a-3p and miR-29a/b/c, both involved in mitochondrial homeostasis in the liver. Beyond epigenetic mechanisms, this sexually dimorphic association may be attributed to differences in lipogenesis and lipolysis [121, 122].

These observations highlight the need for more animal models and larger epidemiologic studies with robust sample sizes that allow for effect modification by sex, which would help to make stronger inferences about the causal links between prenatal exposure and cannabis and the risk of childhood cardiometabolic health.

## Conclusions

Cannabis use during pregnancy is on the rise and may soon surpass tobacco use. Between 2002 and 2016, tobacco use during pregnancy decreased by 40%, whereas the prevalence of cannabis use during pregnancy nearly doubled [123]. This is concerning, as the consequences of cannabis use during pregnancy mirror that of tobacco use during pregnancy: offspring are more likely to be born small, grow rapidly in infancy, and have a higher risk of obesity later in life. While the literature has rapidly expanded since 2014, key gaps in knowledge remain. More data is needed to understand whether these associations are cannabinoid-, timing-, dose-, or sex-specific, which would help to strengthen the biological plausibility and reinforce the need for cannabis cessation efforts in pregnant populations. For the time being, the current recommendations to limit cannabis use during pregnancy should continue. Healthcare providers should have open discussions with pregnant patients about the potential risks of cannabis use during pregnancy and provide evidence-based recommendations for safer alternatives when possible.
